# A Sectional Study: The Relationship between Perceived Social Support and Depression in Turkish Infertile Women

**Published:** 2014-11-01

**Authors:** Kubra Erdem, Serap Ejder Apay

**Affiliations:** Department of Midwifery Erzurum, Ataturk University Faculty of Health Science, Turkey

**Keywords:** Depression, Infertility, Social Support, Sectional Study, Nursing/Midwifery

## Abstract

**Background:**

Studies conducted on infertile women in the literature investigated some features such as depression, anxiety, loneliness, and social support. However, there has been no study examining the relationship between levels of perceived social support and depression in infertile women. Considering this deficiency, the study was conducted to determine the relationship between perceived social support and depression in infertile women. The purpose of this study is to determine the relationship between perceived social support and depression in infertile women.

**Materials and Methods:**

This descriptive and sectional study was conducted between 16 April and 31 October 2012 in *in vitro* fertilisation (IVF) Centre of Fırat University Re- search Hospital. Sampling formula was used in cases when the number of elements in the population was not known to calculate minimum sample size required to be included in the study. A total of 238 women who applied to the relevant centre between the specified dates constituted the sample group of the study. A Questionnaire Form, Beck Depression Inventory (BDI) and the Multidimensional Scale of Perceived Social Support (MSPSS) were used to collect the data. A pilot study was carried out on nine infertile women. As a result of the pilot study, we formed the final version of the Questionnaire Form. The data of these nine women were not involved in the research. The data obtained from the study was assessed using Statistical Package for the Social Sciences (SPSS; SPSS Inc., Chicago, IL, USA) version 15.0. Percentage distribution, mean, t test, one-way analysis of variance (One-Way ANOVA), and Pearson correlation analysis were used to evaluate the data.

**Results:**

The women’s total mean score on the BDI was 12.55 ± 8.07. Scores obtained by women on the MSPSS was 15.75 ± 8.53 for the subscale of friend, 21.52 ± 8.20 for the subscale of family, and 15.62 ± 8.45 for the subscale of significant others. The women’s total MSPSS score was 52.89 ± 21.75.

**Conclusion:**

A significant, negative relationship was found between total BDI score with subscale and total mean scores of MSPSS (r= -0.596, p<0.01). Symptoms of depression decreased as the women’s perceived social support increased.

## Introduction

The family is the smallest union in society that is based on marriage and blood relation and involves relationships between wife, husband, children and siblings (1). The family is responsible for continuing the human race and raising generations appropriate for society’s expectations (2). Having a child is an expected and desired outcome of the conjugal community. As in all societies, marriage is associated with having a child in Turkish society. Therefore, almost all of the married couples or those who intend to get married plan on having a child (3-5). Not all couples, however, are easily able to have a child and may suffer from infertility.

The World Health Organization (WHO) defines "infertility" as the failure of getting pregnant in spite of the couple having unprotected regular sexual intercourse for at least one year (6, 7). The worldwide infertility rate is between 8 and 12% and varies between 10 and 20% in Turkey (6, 8); however, this rate has increased in recent years. This increase is associated with numerous factors such as the change in traditional roles or late marriage and couples’ plans for children. There have also been increases in assisted reproductive techniques, greater access and use of infertility centres, as well as inclusion of infertility diagnosis and treatment in health insurance. From a social perspective, there has been increased accessibility of contraceptive methods; increased social acceptability of infertility; changes in use of alcohol, smoking, and substance abuse; changes in dietary habits, and increased stress and sexually transmitted diseases (7-13).

The effects of infertility on individuals’ emotions are complicated and these effects vary based on the duration of infertility, individuals’ capacities for adaptation, reasons and prognosis of infertility, and emotional and social supports (14-16). Infertility not just a situation about the function of reproduction it also appears as a potential crisis causing social and psychological exposure (17-20). It is estimated that almost 86.8% of infertile women have anxiety and 40.8% have depression. Infertility is a complex life crisis that adversely affects the couples’ social lives, emotional conditions, marriage relations, sex lives, future plans, self-esteem and body images. Infertility should be considered as a bio-psychosocial crisis requiring psychological counselling, which is an integral part of a multidimensional solution (21). Some studies have reported a decrease in the level of depression, anxiety, mental distress, marital violence, and increased rate of pregnancy following psychosocial interventions (14, 22-26). Likewise, infertility treatment is expensive in terms of economy, stressful in terms of emotions and a physically painful process, all of which require adjustment within the couple. Infertile couples might subsequently develop guilt, a sense of worthlessness and depression (2, 19, 20).

Social support, which is a source of coping, is of great importance for the infertile woman to help preserve her physical and mental health. Social support is a valuable coping method that contributes to love, affection, confidence, self-expression, self-knowledge and sense of belonging. Even if it cannot eliminate the stressful situation, it enables individuals to be more optimistic by decreasing their levels of anxiety. It helps individuals in coping with challenging situations and generating new solutions, and decreasing their desperation (4, 27, 28).

Infertility is a condition adversely affecting the woman in terms of biologic, psychological and social aspects. Thus, midwives/nurses who are assigned to support couples in the infertility diagnosis and treatment process have great responsibilities. Infertility nursing is a process that starts in the polyclinic and extends to the operating room; it prioritises psychological and social conditions of couples and includes care during all kinds of medical and surgical treatments (29). During the process of infertility, the general purpose of care is to evaluate the physical, psychological, and social conditions of couples; to determine problems and needs in this field; and thus to provide convenient consultancy and training services (30). The consultancy to be provided to infertile women will positively affect their social support, success of the treatment, and women’s health in the solution of problems.

Based on these important benefits, this study was conducted to determine the relationship between perceived social support and depression in infertile women.

## Materials and Methods

This descriptive and sectional study was conducted in the IVF Centre of a university hospital in the city centre of Elazig (Turkey) between 16 April 2012 and 31 October 2012. The centre where study data were collected was selected because it is the largest unit in the province of Elazýg and renders services to women from surrounding cities and with diverse socio-cultural features.

The population of the study consisted of women who applied to the IVF Centre of Firat University Research Hospital for infertility treatment. The study sample group comprised 238 women who applied to the relevant centre for infertility treatment between the specified dates.

The number of infertile women who accessed the relevant centre for *in vitro* fertilisation (IVF) treatment each year is not known, since the required statistical records have been not regularly kept. Therefore, a sampling formula was used in this situation where the number of elements in the population was not known in order to calculate the minimum sample size required for inclusion in the study. This formula is as follows (31, 32):

n=t^2^*p*q/d^2^
n=Necessary sample size
p=Standard of deviation
q=1- Standard of deviation
t=Z score
d=Margin of error (Confidence interval)
n=(1.96)^2^ *0.15*0.85/(0.05)^2^=195.9216

The number of participants calculated as representing the population was at least 196 individuals. Those women who met the sample criteria and accepted to participate in the study within the period when the researcher was present in the centre were included in the study until the minimum number (196) was attained. This number was reached within 4.5 months. Considering that data loss may occur during the data collection process, the sample size was extended to a total of 238 individuals. The inclusion criteria of the sample group were that women were literate, had been diagnosed with infertility and in the process of having treatment, and they had no serious medical history that threatened life and did not receive treatment because of this reason. The exclusion criteria of the sample group were being not literate, and having a serious medical history.

### Collection of the data

The study data were collected between 16 April 2012 and 31 August 2012. During the data collection process, the following forms were used: "Questionnaire Form" prepared by researchers, "Beck Depression Inventory" (BDI) for evaluation of depression, and the "Multidimensional Scale of Perceived Social Support" (MSPSS) for determination of the perceived social support. The questionnaire and scales were administered by researchers during face-to-face interviews with women who applied to the IVF Centre. Each woman was interviewed in a separate room in the related centre to enable them to answer the questions comfortably. The questionnaire and scales took about 10-15 minutes in total to complete.

### Data collection instruments

#### Questionnaire form

The questionnaire form, which was prepared by researchers based on a literature review, has 26 questions (3, 4, 33-36). The form includes questions that determined the women’s socio-demographic, health, menstruation and infertility characteristics.

### Beck Depression Inventory (BDI)

The 21-item BDI was developed by Beck et al. in 1961 for the purpose of evaluating the physical, emotional, and cognitive symptoms observed during depression. The purpose of the scale is to objectively determine the symptom levels of depression, rather than diagnosing the depression (37, 38). Each item on the scale is scored between 0-3. A higher total score shows the severity of depression symptoms. The validity and reliability study of the Turkish version of the scale was conducted by Hisli in 1988. It indicated that when the BDI score is 17 and above, this enabled the differentiation of depression to be diagnosed with an accuracy of 90% (38). While the criterion-dependent validity was r: 0.75, reliability of split-half method was r: 0.74 and it was reported to be usable in Turkey (38). In this study, the Cronbach’s alpha coefficient for internal consistency was 0.82.

### Multidimensional Scale of Perceived Social Support (MSPSS)

The 12-item MSPSS developed by Zimet et al. in 1988 to subjectively assess the social support was used in this study (39).

The validity and reliability study of the scale was conducted by Eker and Arkar in 1995 (40). The MSPSS contains 12, 7-point, Likert-scaled items which comprise three subscales; family, friends and significant other. Each subscale contains 4 items. In the scale, items 3, 4, 8, and 11 measure the family support; items 6, 7, 9, and 12 measure the friend support; and items 1, 2, 5, and 10 measure the support of significant others. The higher the score obtained from the scale signifies a higher level of perceived social support (39-41). In this study, the Cronbach’s alpha internal consistency coefficients were 0.95 for the subscale of family support, 0.94 for the subscale of the friend support, and 0.91 for the subscale of significant other support. The total Cronbach’s alpha internal consistency coefficient of the scale was 0.94.

### Statistical analysis

The Statistical Package for the Social Sciences (SPSS; SPSS Inc., Chicago, IL, USA) version 15.0 was used to assess the study data. Percentage distributions, mean, t test, one-way analysis of variance (One-Way ANOVA), and Pearson correlation analysis were used to analyse the data.

### Ethical considerations

Before commencing the study, approval was obtained from the Ethics Committee of Ataturk University Faculty of Health Sciences and then written permission was received from the hospital where the study would be conducted. Before starting the data collection process, in order to protect the rights of women included in the study, they were informed about the purpose of the study and that the data would be kept confidential. Any questions from the women were answered and they were given relevant information after the completion of the questionnaire.

Results of the study could be generalised to infertile women in the study group.

## Results

Table 1 and table 2 illustrate descriptive characteristics of women included in the study. Table 2 illustrates the distribution of women’s infertility features and the women’s opinions on infertility.

Table 3 illustrates the distribution of lowest and highest scores obtained from the BDI and MSPSS, along with the women’s mean scores. The women’s total mean score on the BDI was 12.55 ± 8.07. Scores obtained by women on the MSPSS was 15.75 ± 8.53 for the subscale of friend, 21.52 ± 8.20 for the subscale of family, and 15.62 ± 8.45 for the subscale of significant others. The women’s total MSPSS score was 52.89 ± 21.75.

Table 4 illustrates the relationship between BDI with subscale mean scores and total mean scores of the MSPSS. A statistically negative significant relationship is determined between the score of the subscale "friend" in MSPSS and the total score of BDI at the level of p<0.01. There is a statistically negative significant relationship between the score of the subscale "family" in the MSPSS and the total score of BDI at the level of p<0.01. A statistically negative significant relationship was found between the score of the subscale "significant other" in the MSPSS and the total score of the BDI at the level of p<0.01.

According to these results, a statistically negative significant relationship was determined between the scales (r:-0.596, p<0.01). In other words, it was observed that as the women’s perceived social support increases, the symptoms of depression decrease.

Table 5 illustrates some of the women’s socio-demographic characteristics and the comparison of mean scores of the scales of the BDI and MSPSS. No statistically significant difference was determined between the women’s remaining features, except for the mean scores of the BDI according to the women’s educational and income status. The difference between women’s working condition, marriage duration and elapsed time following the diagnosis of infertility and their total mean scores of MSPSS was found to be statistically significant (p<0.05).

**Table 1:** Distribution of women’s socio-demographic and medical characteristics


Characteristics (n = 238)	Mean	Standard deviation

**Age**	31.9	6.2
**Age of marriage (Y)19-30 **	23.7	6.1
**Menstruation duration**	5.9	1.6
**Age of husband (Y)**	35.2	5.9
**Duration of marriage**	8.2	5.8
**Educational status**	**Number**	**%**
Low education level	202	84.9
High education level	36	15.1
**Working condition of women**
Employee (officer+worker)	32	13.4
Unemployed	206	86.6
**Educational status of husband**		
Low education level	167	70.2
High education level	71	29.8
**Occupation of husband**		
Officer	67	28.2
Worker	34	14.3
Self-employed	137	57.5
**Residence where they have lived for the longest period**		
Village-district	80	33.6
Province	158	66.4
**Residence in the city centre where the treatment is conducted**		
Yes	148	62.2
No	90	37.8
**Family Type**		
Nuclear family	213	89.5
Extended family	25	10.5
**Income status**		
High	58	24.4
Low	180	75.6
**Social security**		
Available	205	86.1
N/A	33	13.9
**Mode of marriage**		
Arranged marriage	135	56.7
Love marriage	103	43.3
**Previous depression treatment**		
Yes	20	8.4
No	218	91.6
**Depression treatment after diagnosis of the infertility**		
Yes	19	8.0
No	219	92.0
**Menstrual regularity**		
Regular	154	64.7
Irregular	84	35.3
**Previous reproduction system diseases**		
Yes	89	37.4
No	149	62.6


**Table 2:** Distribution of women’s infertility features


Features (n = 238)	Number	%

**State of having a previous pregnancy**		
Yes	111	46.6
No	127	53.4
**Knowing the reason of infertility**		
Yes	164	68.9
No	74	31.1
**History of treatment**		
Those who had received no treatment before	70	29.4
Hormone therapy	56	23.5
Vaccination*	54	22.7
IVF	58	24.4
**Recent stage of treatment**		
Hormone therapy	17	7.1
Vaccination	65	27.3
IVF	156	65.6
**Obtaining information about the infertility treatment**		
Yes	169	71.0
No	69	29.0
**Source of information (n = 169)**		
Medical staff	136	80.5
Friend, relative, environment	12	7.1
Internet – TV	21	12.4
	**Mean**	**Standard deviation**
**Period to be continued for the infertility treatment (months)**	1.8	1.4
**Elapsed time following the infertility diagnosis (months)**	44.6	47.1


IVF; In vitro fertilisation and *; Following special phases when the ovary is stimulated by drugs and ovulation occurs, sperm are prepared in the laboratory and are inserted into the female genital tracts by means of a catheter.

**Table 3:** Lowest and highest scores of BDI and MSPSS and mean scores of women


	Scales	Lowest and highest scores of scales	Minimum scores of scales	Maximum scores of scales	Mean scores of scales X̅± SD

**BDI**	Total	0–63	0	42	12.55 ± 8.07
**MSPSS**	Friend	4–28	4	28	15.75 ± 8.53
	Family	4–28	4	28	21.52 ± 8.20
	Significant Other	4–28	4	28	15.62 ± 8.45
	Total	12–84	12	84	52.89 ±21.75


BDI; Beck depression inventory and MSPSS; Multidimensional scale of perceived social support.

**Table 4:** Determination of the relationship between the mean scores of MSPSS and BDI


SCALES			BDI total

**MSPSS**	Friend	r	-0.534*
		p	0.000
	Family	r	-0.555*
		p	0.000
	Significant other	r	-0.456*
		p	0.000
	Total	r	-0.596*
		p	0.000


*; P<0.01, BDI; Beck Depression Inventory and MSPSS; Multidimensional Scale of Perceived Social Support.

**Table 5:** Some characteristics of women and comparison of mean scores of BDI and MSPSS


	BDI	Friend	Family	Significant other	Total

**Features**	**X̅ ± SD**	**X̅ ± SD**	**X̅ ± SD**	**X̅ ± SD**	**X̅ ± SD**
**Age**					
19–30	12.23±7.97	16.93±8.28	21.90±8.15	16.72±8.36	55.56±21.59
31 and older	12.80±8.18	14.80±8.64	21.21±8.25	14.73±8.45	50.75±21.72
**Test and p value**	t: 0.53, df: 236, p ˃ 0.05	t: 1.92, df: 236, p ˃ 0.05	t: 064, df: 236, p ˃ 0.05	t: 1.81, df: 236, p ˃0 .05	t: 1.70, df: 236, p˃ 0.05
**Educational status**					
**Low education level**	13.05±8.14	15.37±8.53	21.08±8.36	15.44±8.24	51.91±21.84
High education level	9.69±7.16	17.86±8.32	23.97±6.81	16.61±9.61	58.44±20.63
**Test and P value**	t: 2.32, df: 236, p<0.05	t: 1.61, df: 236, p˃ 0.05	t: 1.95, df: 236, p˃ 0.05	t: 0.76, df: 236, p ˃ 0.05	t: 1.66, df: 236, p ˃ 0.05
**Income state**					
High	10.77±7.40	16.13±9.11	22.79±7.88	16.51±8.83	55.44±21.78
Low	13.12±8.22	15.62±8.35	21.11±8.28	15.33±8.33	52.07±21.74
**Test and P value**	t: 1.93, df: 236 p< 0.05	t: 0.53, df: 236, p˃ 0.05	t: 1.35, df: 236, p˃ 0.05	t: 0.92, df: 236, p˃ 0.05	t: 1.02, df: 236, p ˃ 0.05
**Working condition of women**					
Employee	10.09±6.57	18.06±8.78	25.21±5.83	17.15±9.59	60.43±20.19
Unemployed	12.93±8.23	15.39±8.45	20.95±8.37	15.38±8.26	51.72±21.80
**Test and P value**	t: 1.85, df: 236, p ˃ 0.05	t: 1.65, df: 236, p> 0.05	t: 2.77, df: 236, p< 0.05	t: 1.10, df: 236, p˃ 0.05	t: 3.12, df:236, p˂ 0.05
**Mode of marriage**					
Arranged marriage	12.48±7.63	15.92±8.16	21.49±7.84	15.53±8.01	52.95±20.66
Love marriage	12.64±8.65	15.52±9.03	21.56±8.68	15.73±9.04	52.82±23.20
**Test and P value**	t: 0.15, df: 236, p ˃ 0.05	t: 0.35, df: 236, p˃0.05	t: 0.06, df: 236, p ˃ 0.05	t: 0.18, df: 236, p ˃ 0.05	t: 004, df: 236, p˃ 0.05
**Duration of Marriage**					
1–5	12.51±8.05	16.95±8.39	21.50±7.74	16.69±8.40	55.14±21.04
6–11	11.48±8.23	16.15±8.16	22.97±7.91	15.65±8.81	54.78±21.29
12 and above	14.06±7.80	13.12±8.82	19.58±9.04	13.70±7.82	46.41±22.65
** Test and P value**	F: 1.72, df: 2, p˃ 0.05	F: 3.93, df: 2, p˂ 0.05	F: 2.90, df: 2, p ˂ 0.05	F: 2.32, df: 2, p ˂ 0.05	F: 3.48, df: 2, p ˂ 0.05
**Type of infertility**					
Primary infertility	13.33±8.15	14.97±8.62	21.90±7.91	15.55±8.26	52.43±20.99
Secondary infertility	11.65±7.92	16.63±8.37	21.09±8.52	15.70±8.70	53.43±22.67
**Test and P value**	t: 1.59, df: 236, p˃ 0.05	t:1.50, df: 236, p ˃ 0.05	t: 0.76, df: 236, p˃ 0.05	t: 0.13, df: 236, p˃ 0.05	t: 0.35, df: 236, p˃ 0.05
**Infertility period (month)**					
1-12	12.97±7.62	14.87±8.34	21.41±8.34	15.07±8.27	51.36±21.47
13–24	9.92±7.15	18.69±8.59	24.07±5.86	18.61±8.09	61.38±18.84
25–36	12.45±7.77	15.37±9.08	18.66±9.30	13.95±9.70	48.00±24.71
37 and above	13.38±8.74	15.26±8.36	21.21±8.48	15.15±8.25	51.63±21.77
**Test and P value **	F: 1.91, df: 3, p˃ 0.05	F: 2.08, df: 3, p˃ 0.05	F: 2.41, df: 3, p ˃ 0.05	F: 2.30, df: 3, p˃ 0.05	F: 2.83, df: 3, p˂ 0.05


BDI; Beck depression inventory and MSPSS: Multidimensional scale of perceived social support.

## Discussion

Diagnosis and treatment approaches used for infertile couples may hinder their coping skills and social support resources; consume their physical and emotional energy; cause sexual dysfunctions, depression, anxiety, and loneliness; and damage the couple’s relationship (14, 26, 33-36). The results obtained from this study, conducted in order to determine the relationship between the perceived social support and depression in infertile women, are discussed in line with the relevant literature.

Examining the women’s mean scores from the MSPSS and BDI (Table 3) determined that their BDI mean score was lower. This result is similar to that found in other studies where the mean score of depression is low (3, 14, 27, 31, 42-44). In the study conducted by Gurbuz, the women’s mean score of depression was 21.11 ± 5.74. We determined that the women’s total mean score on the MSPSS was close to the highest score that could be obtained from the scale where the highest perceived social support was from subscales "family", "friend" and "significant other", respectively (34). In Kus’s study, the subscale mean scores and total mean scores of MSPSS also showed a similarity with the results of this study (4). A controlled study conducted by Upkong and Orji with 208 women in Nigeria revealed that receiving no support from the husband increased women’s depression and anxiety scores (45). Similarly, in the study conducted by Matsubayashi et al. (44) with 101 infertile women in Japan, the researchers determined that women’s anxiety and depression levels were very high and this was associated with lack of support from their husbands. In the light of such information, it could be stated that when social support meets individuals’ expectations, especially the support of family, it enables individuals to cope with life’s problems by showing a positive effect in terms of morale and coping.

After examining the relationship between the BDI total score with subscale mean scores and total mean scores of MSPSS shown in table 4, a statistically negative significant relationship was found between the scales at the level of p<0.01. It was determined that as the women’s perceived social support increases, symptoms of depression decrease. Social support is a predictive factor for depression (43). The studies conducted with infertile women concluded that lack of social support caused a higher rate of anxiety and depression symptoms in infertile women (43-45). It is also reported that the lack of husband and his family’s support results in the deterioration of mental health and depression (15, 45).

On examination of the BDI mean scores and educational status of women according to some of their features shown in table 5, it was observed that those with a high educational level (university graduate) had fewer symptoms of depression. As the educational level increases, it becomes easier for women to have a better economic status, social security and access to knowledge. Women who have access to full information might experience less worry, obscurity, and anxiety. Thus, it is possible to assert that women with higher educational levels have lower BDI scores. In the study conducted by Pinar, women’s mean score of depression was 26.79 ± 10.90 (26).

Considering the income status of women, it is thought that those with a lower income status have a higher BDI mean score; in other words, those with a poor income status are negatively affected in terms of experiencing depression. In line with result of this study, there are some studies asserting that as the income status increases, the level of depression decreases (3, 4, 6, 26).

Examining the women’s MSPSS mean scores also showed that the difference between women’s working conditions with total mean score of MSPSS and mean score of its subscale "family" was found to be statistically significant (p<0.05). As the women’s educational level increases, they are enabled to have a regular job and income; access positive information, attitudes and behaviours in terms of health; and also ensure their families attain positive information, attitudes and behaviours on this subject; it could, therefore, be asserted that the women’s perception of social support is also affected positively.

The difference between marriage duration of women with total mean score of the MSPSS and mean score of its subscale "friend" was found to be statistically significant (p<0.05). As the marriage age increases, the perceived social support decreases-similar results were also found in the study conducted by Eren (27).

The difference between women’s infertility periods and total mean scores of the MSPSS was found to be statistically significant (p<0.05). Similarly, Kus’s study also observed that the difference between the elapsed time following the diagnosis of infertility and total mean scores of MSPSS was statistically significant (4).

## Conclusion

In consequence of this study, it is observed that as the women’s perceived social support decreases, their mean scores of depression increase. The recommendations made in line with results of the study are:

Informing midwives and nurses about the problems experienced by infertile women and interventions aimed at these problems through in-service training programs is important.

Enabling midwives and nurses to examine the social support mechanisms of women diagnosed with infertility, helping them to use the support involved in family and other social support systems positively, as well as making interventions that strengthen the social support systems of individuals with insufficient support, will improve overall care outcomes.

Evaluating the infertile woman both gynaecologically and psychologically, and providing her with contact with a psychologist or psychiatrist when required, can improve psychological health.Infertile women would benefit from close follow up in terms of depression risk.

By conducting comparative studies in groups where pregnancy is achieved or not achieved as a result of the treatment would help to determine the explicit effect of the perceived social support on depression in infertile women.

## References

[1] Family.

[2] Tasci KD, Ozlem S (2007). University School for Health Sciences students’ opinions about infertility. TAF Prev Med Bull.

[3] Oguz HD (2004). Mental health in women undergoing infertility treatment infertility, marriage, relationships, and the effects of sexual life.Presented for M.Sc..

[4] Kus C (2008). Determination of the life quality and perceived social support of women in infertile case.Presented for M.Sc..

[5] Ejder S (2008). Family planning methods engaged-couples thought to use after marriage. Journal of Anatolia Nursing and Health Sciences.

[6] World Health Organization (2002). Medical, ethical and social aspects of assisted reproduction.

[7] Atasu T, Sahmay S (2001). Gynecology.

[8] Arici A, Attar E, Balaban E, Buyru F, Çolgar U (2006). Reproductive endocrinology and infertility.

[9] Kavlak O, Saruhan A (2002). A study on determination the loneliness level in infertile women and to assess the factors that effect the loneliness level. Ege Journal of Medicine.

[10] Taskin L (2011). Maternity and women’s health nursing.

[11] Goldman MB, Missmer SA, Barbieri RL, Goldman MB, Hatch MC (2000). Infertility. Woman and health.

[12] Speroff L, Fritz MA (2007). Clinical gynecologic endocrinology and infertility.

[13] Aksu T, Demirtas E, Gunalp GS, Tuncer ZS (2004). Assisted reproductive techniques. Diagnosis and treatment of obstetrics and gynecology.

[14] Ramezanzadeh F, Aghssa MM, Abedinia N, Zayeri F, Khanafshar N, Shariat M (2004). A survey of relationship between anxiety, depression and duration of infertility. BMC Women’s Health.

[15] Karlidere T, Bozkurt A, Yetkin S, Doruk A, Sutcigil L, Ozmenler KN (2007). Is there a gender difference in infertile couples without an axis one psychiatric disorder in the context of emotional symptoms, social support and sexual function. Turkish Journal of Psychiatry.

[16] Dilek N (2009). Determination of the emotional reactions of the couples who have been treated with assisted reproductive techniques.Presented for M.Sc..

[17] Holter H, Anderheim L, Bergh C, Möller A (2006). First IVF treatment--short-term impact on psychological well-being and the marital relationship. Hum Reprod.

[18] Ungerleider J, Rothchild TC, Nichols L (2008). The Boston IVF: handbook of infertility.

[19] Ozcelik B, Karamustafalioglu O, Ozcelik A (2007). The psychological and psychiatric aspects of infertility. Anatolian Journal of Psychiatry.

[20] Alibasoglu H (2010). Infertility emotional symptoms, marital adjustment, and sexual function in the context of gender difference.Presented for M.Sc..

[21] Van den Broeck U, Emery M, Wischmann T, Thorn P (2010). Counselling in infertility: individual, couple and group interventions. Patient Educ Couns.

[22] Chan CH, Ng EH, Chan CL, Ho Chan TH (2006). Effectiveness of psychosocial group intervention for reducing anxiety in women undergoing in vitro fertilization: a randomized controlled study. Fertil Steril.

[23] Chen TH, Chang SP, Tsai CF, Juang KD (2004). Prevalence of depressive and anxiety disorders in an assisted reproductive technique clinic. Hum Reprod.

[24] Cousineau TM, Lord SE, Seibring AR, Corsini EA, Viders JC, Lakhani SR (2004). A multimedia psychosocial support program for couples receiving infertility treatment: a feasibility study. Fertil Steril.

[25] Domar AD, Clapp D, Slawsby EA, Dusek J, Kessel B, Freizinger M (2000). Impact of group psychological interventions on pregnancy rates in infertile women. Fertil Steril.

[26] Pinar G, Zeyneloglu HB (2012). Quality of life, anxiety and depression in turkish women prior to receiving as¬sisted reproductive techniques. Int J Fertil Steril.

[27] Eren N (2008). The impact of the levels of perceived social support in infertile couples on stress related with infertility and marital adjustment.Presented for Ph.D..

[28] Ardahan M (2006). Social support and the nursing. Journal of Anatolia Nursing and Health Sciences.

[29] Kilic M, Apay Ejder S, Kızılkaya Beji N (2011). Infertility and culture. Journal of Florence Nightingale School of Nursing.

[30] Yanıkkerem E, Kavlak O, Sevil U (2008). Infertile couple’s problems and nursing approach. Journal of Anatolia Nursing and Health Sciences.

[31] Akturk Z, Acemoglu H (2010). Statistical research and practice for health care professionals.

[32] Determining sample size: how to ensure you get the correct sample size.

[33] Benli S (2010). Depression and anxiety among infertile women and related factors.Presented for M.Sc..

[34] Gurbuz SK (2007). The effect of marital adjustment and depressive mood in infertile couples on the results of in vitro fertilization-embryo transfer (IVF-ET).Presented for M.Sc..

[35] Akyuz A, Inanc N, Pabuccu R (1999). Spouses in vitro fertilization unit will be based on experience and requirements for the planning of nursing activities. Gulhane Medical Journal.

[36] Lee TY, Sun GH, Chao SC (2001). The effect of an infertility diagnosis on the distress, marital and sexual satisfaction between husbands and wives in Taiwan. Hum Reprod.

[37] Beck AT, Ward CH, Mehdelson M, Mosk J, Erbaugh J (1961). An inventory for measuring depresssion. Arch Gen Psychiatry.

[38] Hisli N (1989). Beck depression inventory for university students validity and reliability. Turkish Journal of Psychology.

[39] Zimet GD, Dahlem NW, Zimet SG, Farley GK (1988). The multidimensional scale of perceived social support. J Pers Assess.

[40] Eker D, Arkar H (1995). Perceived social support: psychometric properties of the MSPSS in normal and pathological groups in a developing country. Soc Psychiatry Psychiatr Epidemiol.

[41] Eker D, Arkar H, Yaldiz H (2001). Factorial structure, validity, and reliability of revised form of the multidimensional scale of perceived social support. Turk Psikiyatri Derg.

[42] Akyuz A (2001). Nursing in adaptation to IVF failure.Presented for Ph.D..

[43] Yilmaz I (2006). The determinants of depression and anxiety in Turkish infertility patients: social support, sex role orientation, infertility causality, and self-esteem.Presented for M.Sc..

[44] Matsubayashi H, Hosaka T, Izumi S, Suzuki T, Kondo A, Makino T (2004). Increased depression and anxiety in infertile japanese women resulting from lack of husband’s support and feelings of stress. Gen Hosp Psychiatry.

[45] Upkong D, Orji E (2006). Mental health of infertile women in Nigeria. Turkish Journal of Psychiatry.

